# What Persons with Chronic Health Conditions Need to Maintain or Return to Work—Results of an Online-Survey in Seven European Countries

**DOI:** 10.3390/ijerph15040595

**Published:** 2018-03-26

**Authors:** Nicole Foitzek, Carolina C. Ávila, Ivana Ivandic, Črtomir Bitenc, Maria Cabello, Sonja Gruber, Matilde Leonardi, Amalia Muñoz-Murillo, Chiara Scaratti, Beata Tobiasz-Adamczyk, Anastasia Vlachou, Eva Esteban, Carla Sabariego, Michaela Coenen

**Affiliations:** 1 Chair for Public Health and Health Services Research, Research Unit for Biopsychosocial Health, Department of Medical Information Processing, Biometry and Epidemiology (IBE), Ludwig-Maximilians-Universität (LMU), 81377 Munich, Germany; nicole.foitzek@web.de (N.F.); ivana.ivandic@med.lmu.de (I.I.); eva.esteban@med.lmu.de (E.E.); carla.sabariego@med.lmu.de (C.Sa.); 2 Department of Psychiatry, Universidad Autónoma de Madrid and Institute of Health Carlos III, CIBER of Mental Health (CIBERSAM), 28038 Madrid, Spain; carolina.avila@uam.es (C.C.Á.); maria.cabello@uam.es (M.Ca.); 3 Development Center for Vocational Rehabilitation, University Rehabilitation Institute Republic of Slovenia, 1000 Ljubljana, Slovenia; crtomir.bitenc@ir-rs.si; 4 Disability and Diversity Studies, Carinthia University of Applied Science (CUAS), 9020 Klagenfurt, Austria; s.gruber@fh-kaernten.at; 5 Neurology, Public Health and Disability Unit; Neurological Institute Carlo Besta IRCCS Foundation, 20133 Milan, Italy; matilde.leonardi@istituto-besta.it (M.L.); chiara.scaratti@istituto-besta.it (C.Sc.); 6 Research Unit, Parc Sanitari Sant Joan de Déu, Universitat de Barcelona, 08830 Sant Boi de Llobregat, Barcelona, Spain; a.munoz@pssjd.org; 7 Chair of Epidemiology and Preventive Medicine, Jagiellonian University Medical College, 31-034 Krakow, Poland; mytobias@cyf-kr.edu.pl; 8 Department of Special Education, University of Thessaly, 38221 Volos, Greece; anavlachou@uth.gr

**Keywords:** employment, chronic disease, work, needs, qualitative research

## Abstract

Chronic health conditions represent the major share of the disease burden in Europe and have a significant impact on work. This study aims to: (1) identify factors that have a negative or positive impact on the work lives of persons with chronic health conditions; (2) explore the needs of these persons to maintain a job or return to work and (3) compare these results with respect to these persons’ occupational status. An online survey was performed in seven European countries. Open-ended survey questions were analyzed using qualitative methods. In total, 487 participants with six chronic health conditions participated. The majority of participants named work-related aspects (such as career development, stress at the workplace, work structure and schedule as well as workload), support of others and attitudes of others as being the factors positively and negatively impact their work lives the most. Our study shed light on the importance of changing the attitudes of supervisors and co-workers to counteract stigmatization of persons with chronic health conditions in the workplace. In conclusion, this study provides a basis for developing new strategies of integration and reintegration at work for persons with chronic health conditions in European countries.

## 1. Introduction

Chronic health conditions contribute to the major share of the disease burden [1,2,3] and are the leading cause of mortality and morbidity in Europe [4]. They are defined as health problems which require ongoing management and treatment over years or even decades [5]. For most people, facing such long-term health conditions is a challenging experience, affecting everyday life and participation.

For persons of working age, living with a chronic health condition might impact their ability to find or maintain employment, and consequently, threaten their financial security and that of their families. Approximately one out of three employees has at least one chronic health condition and 42% of them report that their conditions haves an impact on their work life, as Steadman and colleagues reported for the UK [6]. Experiencing negative impacts on work life in European countries because of chronic health conditions results in an increased risk of loss of income and poverty, social exclusion and severe material deprivation [7]. The employment rate of persons with restrictions in their work life due to health conditions was 30 percent less compared to people with no such restrictions, according to data of the ad hoc module of the 2015 EU Labour Force Survey [8]. Persons with chronic health conditions have more difficulties in finding and keeping a job compared to people without health conditions as Kessler and colleagues showed for the U.S. [9].

Although persons with chronic health conditions might experience restrictions regarding work, being able to maintain and return to work remains very important for their well-being. Waddell and Burton conducted an extensive review on the relationship of work on health and well-being [10]. The authors provide evidence that engaging in work life provides financial independence and psychosocial well-being; it is a source of identity and contributes to peoples’ social status. In contrast, as a consequence of unemployment, individuals experience poor physical and mental health and need more medical consultations [10]. Persons returning to work experience the benefit of work such as improved self-esteem, physical function, a stable financial situation and reduced psychological distress. Employment is also a source of meaning in peoples’ lives. In contrast, short or long-term unemployment due to health conditions may influence a person’s values, as it prevents the “action or “doing” that (allows) meaning (to be) realized in our lives” [11].

The ability to maintain or return to work does not depend, however, only on the health conditions but is importantly influenced by a person’s physical, social, attitudinal and political environment. Adapting the working environment to the needs of persons with chronic health conditions is a task that employers and politicians have to consider also in light of an aging (work) population and an increase of chronic health conditions European countries are faced with during the next decades. However, knowledge of factors that have a positive and negative impact on work life as well as of the work-related needs of persons with chronic health conditions is necessary to identify or develop interventions that can support them maintaining or returning to work after a long absence. Up to now evidence to answer is missing. Therefore, this study aims to: (1) identify factors that have a negative or positive impact on work lives of persons with chronic health conditions; (2) explore the needs of these persons to maintain or return to work and (3) compare these results with respect to these persons’ occupational status.

## 2. Materials and Methods

### 2.1. Design

This study was carried out within the scope of the EU-funded project “Participation in Healthy Workplaces and Inclusive Strategies in the Work Sector” (PATHWAYS; grant agreement n. 663474) [12]. This 3-year project aims to identify strategies of integration and reintegration for persons with chronic health conditions in Europe, to evaluate their effectiveness and to assess the specific employment-related needs of these persons. The expected final result of the PATHWAYS project is to develop European guidelines to support the implementation of effective professional integration and reintegration strategies for people with chronic health conditions. The project consortium is made up of partners from ten European countries, namely Austria, Belgium, Czech Republic, Germany, Greece, Italy, Norway, Poland, Slovenia and Spain. Within the project, an online survey mainly assessing quantitative data was carried out to get first-hand evidence from persons with selected chronic health conditions about their needs to maintain or return to work. To this survey a qualitative part including open-ended questions was added by seven of the ten partners, namely Carinthia University of Applied Sciences (Klagenfurt, Austria), Fondazione IRCSS Istituto Neurologico Carlo Besta (Milan, Italy), Ludwig-Maximilians-Universität (Munich, Germany), Parc Sanitari Sant Joan de Déu (Barcelona, Spain) and University Autónoma de Madrid (Madrid, Spain), University Jagiellonski (Krakow, Poland), University of Thessaly (Volos, Greece) and University Rehabilitation Institute, Republic of Slovenia (Ljubljana, Slovenia). In this paper, we focus on the analysis of the qualitative part of the survey.

All study-related documents were submitted to the Ethic Committees of the study centres prior to the commencement of the study. All study centres received ethical approvals from their respective Ethic Committees (the registration number of the ethical approval at LMU is 456-16). The study was carried out according to the Declaration of Helsinki, 1996.

### 2.2. Participants

Participants in the survey had to fulfil the following inclusion criteria: (1) being of working age (at least 18 years old) and (2) having at least one of the following chronic health conditions: back and neck pain, chronic obstructive pulmonary diseases (COPD), depression, diabetes mellitus (DM), ischaemic heart disease (IHD) and migraine/headache disorders. These six chronic health conditions were selected within the scope of the PATHWAYS project based on the leading causes of years lost due to disability (YLD) in Europe [3].

### 2.3. Material

An English template questionnaire was set up including closed and open-ended questions for assessing quantitative and qualitative data, respectively. Closed questions address the following data: age, gender, type of occupation according to the International Standard Classification of Occupations (ISCO) [13], occupational status (employed, not employed), working sector (private, public, non-profit sector) and the chronic health condition(s) (back and neck pain, COPD, depression, DM, IHD, migraine/headache disorders). Additional chronic health conditions could be reported in a free text field. The participant’s experiences of (1) factors that negatively and positively impact their work lives and (2) needs to maintain or to return to work were assessed by using open-ended questions (see Table 1). The open-ended questions were developed and approved by all partners involved in the study. Partners translated the questionnaire into their national languages and set up online surveys in the respective languages on a Google platform.

### 2.4. Data Collection and Data Preparation

The survey was carried out between August and October 2016. Patient organizations at national level focusing on the six chronic health conditions were contacted via email to recruit participants. Patient organizations were identified using a stakeholder list which was developed during the PATHWAYS project. The email included a link to the survey and further information about the study, along with the request to contact members of the patient organizations and to inform them about the survey. Distribution of the link and information were the responsibility of the patient organizations; some of them published announcements on their websites, others included information in regular newsletters or contacted members via e-mail.

The data was simultaneously collected in seven countries, namely Austria, Germany, Greece, Italy, Poland, Slovenia and Spain. After completion of the data collection, the answers to the open-ended questions were translated from the original languages into English by the responsible study coordinators in the respective countries. An aggregation of the translated answers was undertaken and data was reviewed for comprehensibility and completeness. If needed, discrepancies and ambiguities were clarified with the respective study coordinators.

Health conditions others than the six selected health conditions were categorized to disease-specific groups using the study participants’ self-reported diagnoses (e.g., anxiety disorder, breast cancer, multiple sclerosis, epilepsy). As some of these health conditions did not meet the criteria for chronic health conditions, (e.g., slipped disc, difficulties with concentration), we first excluded them and then summarized the chronic health conditions into broader categories (e.g., mental health conditions, cancer, neurological disorders) to facilitate the reporting of the results.

### 2.5. Data Analysis

We performed descriptive data analysis of the quantitative data in SPSS (IBM, Armonk, NY, USA) [14] by calculating absolute and relative frequencies. Qualitative data analysis of the open-ended questions was carried out using the content-related coding methodology according to Mayring’s inductive content analysis [15]. In this analysis, coding categories are directly extracted from the data [15,16]. We first coded the participants’ original answers and created categories. In addition to the coding procedure proposed by Mayring we used these categories to build higher level categories and overarching themes, to build broader units of data analysis and ensure clarity and readability of the results.

In the beginning, we ran a pre-test to agree on the coding procedure and level of specification of categories among the researchers involved in this exercise in which 10 percent of the answers were double coded. A structured discussion about the categories, higher-level categories and themes was conducted. In case of disagreement, a third opinion was taken into account. After completing the pre-test, the original answers were read several times for complete understanding. Regular ballot meetings with the involved researchers were held to finalize the coding exercise.

**Figure 1:**
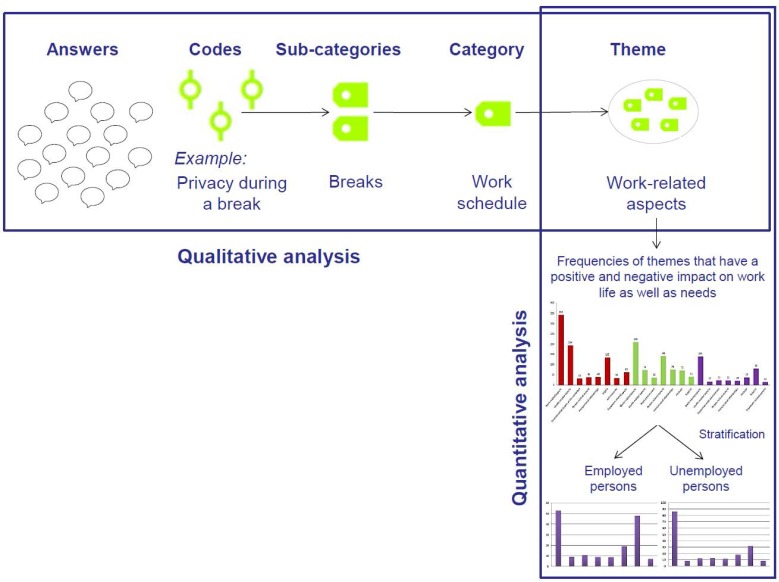
Scheme of qualitative and quantitative analysis; an example of the data analysis.

The codes were categorized based on similar answers and areas. Figure 1 shows the procedure of building categories in the qualitative analysis. Most answers contained several enumerations so two or more codes were used per one response. To facilitate coding, categorization and organization of data the software MAXQDA [17] (VERBI Software: Berlin, Germany) was used.

After having finalized the qualitative analysis, absolute and relative frequencies of the extracted themes were calculated for the total sample and stratified by occupational status (employed and unemployed) (see Figure 1).

To enhance the readability of the results section, factors with a negative or positive impact on work life as well as needs are written in italic letters; themes are enclosed by quotation marks and categories begin with a capital letter.

## 3. Results

### 3.1. Description of Study Participants

**Figure 2:**
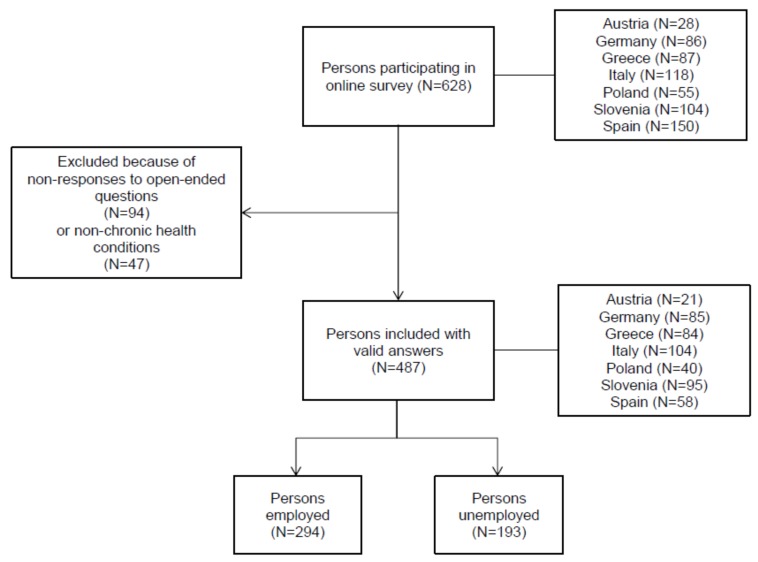
Flowchart of the study selection process.

In total, 628 persons participated in the qualitative part of the survey. Of these, 487 persons answered the open- ended questions. The following results were based on these persons (see Figure 2). The age of the participants ranged from 20 and 67 years, with a median age of 46 years. The participants’ detailed characteristics are presented in Table 2. Two hundred eighty-two study participants (57.9%) reported one of the six chronic health conditions, 185 (38.0%) reported two and 20 participants (4.1%) reported three or more of the six chronic health conditions.

### 3.2. Factors Having a Positive or Negative Impact and Needs

**Figure 3:**
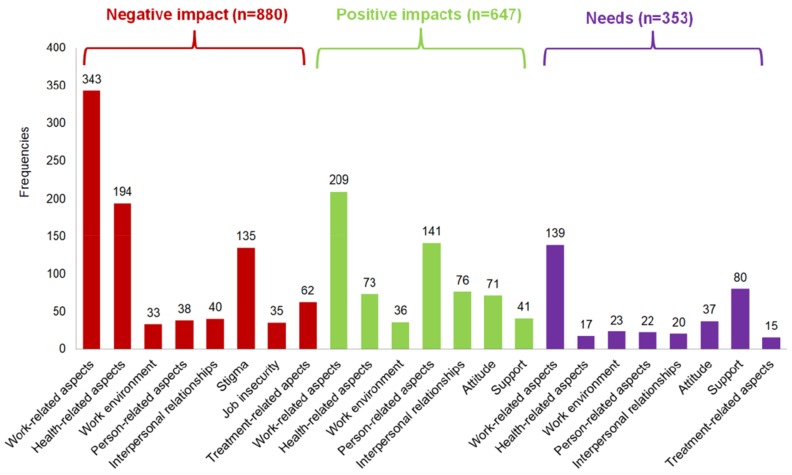
Frequencies of codes in the different themes for the *Factors having a negative impact* and *Factors having a positive impact* on work life, as well as the *Needs* to maintain a job or return to work.

In total, 428 persons (87.9%) reported on *Factors having a negative impact* and 407 persons (83.6%) on *Factors having a positive impact* on work life. Only 191 (39.2%) reported on *Needs* that would be helpful to maintain a job or return to work. From all answers we identified 1880 codes (*n* = 880 *Factors having a negative impact*; *n* = 647 *Factors having a positive impact*; *n* = 353 *Needs*) (see Figure 3). We built 101 categories based on the 1880 codes, and from these ten themes were formed (see Table 3).

#### 3.2.1. Factors Having a Negative Impact on Work Life

As shown in Figure 3, the most frequently identified theme of the *Factors having a negative impact* on work life was “Work-related aspects” followed by “Health-related aspects” and “Stigma”.

- “Work-related aspects”: Categories of this theme are Career development, Food at the canteen Home-work interface, Stress, Workplace, Work structure, Work schedule as well as Workload and work pace. Career development resulted from the participants’ view of having difficult advancement opportunities when having a chronic health condition.

“Difficult advancement opportunities”

(54, female, Germany, employed, migraine/headache disorders)

*“Lack of real opportunities”*

(50, female, Poland, employed, depression; migraine)

“Lack of development”

(28, male, Poland, employed, DM)

The category Stress was one of the most frequently named *Factors having a negative impact* on work life, and more specifically stress-related experiences at their workplace, with a focus on the lack of performance at work.

“Too much stress load”

(58, male, Slovenia, employed, COPD)

“Stress; periods, when I cannot even rest because of my breathing problems and I have to work “like crazy””

(62, male, Slovenia, employed, COPD)

*“That I couldn’t reveal the fact that I was fighting with depression which resulted in being under stress and pressure”*

(49, female, Greece, unemployed, depression)

*“Daily stress in order to be able to complete all my duties with responsibility and consistency”*

(34, female, Greece, employed, depression)

A fairly important category experienced by the study participants in the theme “Work-related aspects” was Workload and work pace. Facing problems in the workplace such as high pressure, responsibility and workload concerned the participants the most.

*“Too many duties”*

(28, male, Poland, unemployed, DM)

*“Incapable of achieving the high standards, set by employer”*

(43, male, Slovenia, employed, migraine/headache disorders)

*“When sometimes I have to work for many hours without resting because the company is mine and I have to be there all the time”*

(61, male, Greece, employed, COPD)

*“I have to perform my job in the same time as my colleagues, but at the same time I have to manage my disease”*

(31, female, Italy, employed, DM)

Also the theme Work structure including aspects such as face-time at work, breaks and time schedule was reported as *Factors having a negative impact* on the work life of persons with chronic health conditions.

*“Having no breaks in case of worsening of my disease”*

(58, female, Poland, unemployed, depression)

*“Overload resulting from “duty” to sit at the desk even when there was no work to be done”*

(31, female, Poland, employed, depression)

*“Start to work early in the morning because I often have nocturnal and early morning crises”*

(51, female, Italy, employed, migraine/headache disorder; back and neck pain)

- “Health-related aspects”: The theme “Health-related aspects” include issues mainly focusing on impaired body functions as defined by the International Classification of Functioning, Disability and Health (ICF) [18] and diagnoses. With regard to impaired body functions, study participants stressed problems with concentration, emotional functions and disfigurement of the body or body parts.

“Anxiety that I can’t discharge my duties at work because of my mood”

(52, female, Poland, unemployed, depression)

*“Anxiety whenever I could not go to work because of the intensity of my symptoms—something that could result in my dismissal”*

(49, female, Greece, unemployed, depression; IHD)

- “Stigma”: The study participants reported on Stigmatization as one of the *Factors having a negative impact* that is hard to deal with at their workplace. The experiences of discrimination reported by the persons involved in our study are related to a lack of knowledge or understanding of their chronic health conditions. Stigma, as defined by Thornicroft and colleagues [19], is specified in the three parts, Knowledge, Attitudes and Behavior. The study participants often named problems with colleagues or supervisors due to a lack of knowledge and understanding that led to conflicts, discrimination and maladjustment.

*“Lack of understanding of chronic health condition by supervisors and colleagues”*

(36, female, Germany, employed, depression)

*“Always being the one with the “sickness bonus”*

(22, female, Austria, employed, DM)

*“I was always referred to as “not fully deployable, fit or able to perform”*

(67, male, Austria, unemployed, COPD; IHD)

*“Misunderstanding from managers and ignorance about the disease”*

(58, female, Slovenia, unemployed, back and neck pain)

*“The fact that most of my colleagues and supervisors do not pay attention to my health situation and they continue to load me with more responsibilities, while they see that I cannot keep up”*

(34, female, Greece, employed, depression)

#### 3.2.2. Factors Having a Positive Impact on Work Life

The three most important themes from the perspective of persons with chronic health conditions are “Work-related aspects”, “Person-related aspects” and “Interpersonal relationships” as shown in Figure 3.

- “Work-related aspects”: One of the categories derived from this theme is having a standard salary. Also the aspect of having enough income to finance treatments or medications was mentioned by the study participants as a *Factor having a positive impact* on their work lives.

*“With that income I can afford quite a lot that contributes to my own and my family’s physical, mental and emotional well-being”*

(50, male, Austria, employed, DM)

*“Certainly the fact that I have a regular salary every month and I can pay doctors and medications, makes me feel safe”*

(34, female, Greece, employed, depression)

The Employment status was another important aspect mentioned by the participants. Having a permanent contract as a part-time job or a stable position was one of the *Factors having a positive impact* on the work lives of the study participants.

*“Many years in the same company and a stable position”*

(50, female, Poland, employed, depression, migraine/headache disorders)

“I have permanent contract”

(50, male, Italy, employed, COPD)

*“I have obtained a part-time work”*

(51, female, Italy, employed, migraine/headache disorders)

With regard to job-related structure, Autonomy, Responsibility and the Variety of work were mentioned by the participants.

*“My own design of the working day”*

(52, female, Germany, employed, migraine/headache disorders)

*“A job with diverse tasks, with external operations (not always inside the office)”*

(37, female, Spain, unemployed, DM)

Other categories reported by the participants were the Home-work interface including aspects such as a short commute to the workplace or having less stressful situations at the workplace. Another *Factor having a positive impact* on the work lives of persons with chronic health conditions was having a good (working and interpersonal) atmosphere between colleagues and supervisors. A Work schedule with flexible or regular working hours, breaks and working patterns to enable a structured daily routine was also important for the respondents.

*“To be able to take a break or leave when I need to (flexible hours)”*

(62, male, Greece, employed, COPD)

*“A daily routine meant that time was passing more easily, and I was not thinking of the difficulties”*

(54, female, Greece, unemployed, COPD)

- “Person-related aspects”: This theme is an important factor for coping with a chronic health condition. Most of the answers are categorized in Thoughts and beliefs and Motives such as the person’s needs and goals. Another aspect of this theme having a positive impact on work life is Feelings and emotions of persons and their position in the social context. Almost half of the “Person-related aspects” deal with codes coming from the category Thoughts and beliefs. Most of them are resources that help to deal with a chronic health condition such as “being creative”, “being calm”, “positive thinking”, “self-discipline” and “self-esteem” to name just some of them.

*“I am motivated every day to perform as well as a healthy employee”*

(63, female, Austria, employed, COPD; migraine/headache disorders)

*“Creativity and continuous mind functioning”*

(49, female, Greece, unemployed, depression)

Motives that include the needs and goals of people are also identified as “Person-related aspects”. An often named goal of our study participants is self-fulfillment, including having a normal life or a successful personal life. Likewise, leisure pursuits such as music, literature and physical activity are important resources for people dealing with chronic health conditions and are experienced as Factors having a positive impact on work life.

*“Partial possibility of self-fulfillment (it’s better than sitting at home and having nothing to do)”*

(28, male, Poland, employed, DM)

*“Physical work activity has been beneficial”*

(61, male, Spain, unemployed, DM)

- “Interpersonal relationships”: Relationships with colleagues and supervisors play an important role in the integration in the workplace. The study participants expressed their perception of being a part of the team or company. *Factors having a positive impact* that were often named by the study participants included Communication with people and having a Good atmosphere at the workplace.

*“Meeting a lot of people and the opportunity to earn for myself”*

(28, male, Poland, employed, DM)

*“Positive and balanced relations with my co-workers”*

(31, female, Slovenia, employed, DM)

*“Something positive in general, was the communication with my colleagues”*

(43, male, Greece, unemployed, IHD)

#### 3.2.3. Needs to Maintain or Return to Work

Responses to the *Needs* of persons with chronic health conditions mainly focus on “Work-related aspects” and “Support” at the workplace.

- “Work-related aspects”: Nearly half of the *Needs* of people with chronic health conditions deal with “Work-related aspects”. One of these “Work-related aspects” was Work schedule; the study participants stressed the need, variously, to work without interruption, have flexible working models, working hours and fixed or flexible breaks.

*“Offer flexible working models and accept needs”*

(53, male, Germany, employed, depression)

Also the Work pace was mentioned as a need that should be modified by reducing the workload for less stress and less pressure. According to the statements of our study participants, the workplace should be adapted to the needs of people with health problems: Participants would favor the possibility of home office or transportation to and from work. As a factor of Workplace adaptation the job content should also be adapted to the needs of persons with chronic health conditions. For instance, helpful modifications could be less responsibility, having a choice, no lifting of heavy weights or no external working tasks.

*“Consideration of individual needs at the workplace and from the employment office regarding professional choice”*

(37, female, Germany, unemployed, depression; migraine/headache disorders)

*“The opportunity to inform the employer about illness and help in the adaptation of the working environment”*

(21, female, Poland, unemployed, depression)

*“Flexible workplaces for people with chronic health conditions”*

(46, female, Germany, employed, migraine/headache disorder; depression)

Aspects concerning the Organizational culture like teamwork, less fluctuation, more days of annual holidays and proper guidance should be part of corporate culture. Another important need is the financial aspect of income and having the possibility of early retirement or changing the terms of the employment contract.

- “Support”: The study participants’ answers concerning different “Support” measures are divided into three categories: Financial support, Support of others such as coworkers and supervisors and Service-related support. A great demand for “Support” was identified in the field of Financial support concerning the support from the state or health insurance systems. It was also important for the participants not to lose their benefits when finding a new job.

*“Not to lose the benefits in case of finding a job”*

(42, female, Greece, unemployed, COPD)

*“Recruitment could come from an administration benefit for the disabled”*

(48, female, Spain, unemployed, migraine/headache disorder; depression)

The study participants stressed their Need for returning to work or to maintain their work while facing problems with their chronic health conditions and getting support for this. It was mentioned as helpful from a service-related view to have a variety of portals or institutions for job hunting in order to increase the number of jobs for people with chronic health conditions. Another need with regard to service-related support that was mentioned by the participants was psychological support from professionals at the workplace.

*“A portal of institutions employing chronically ill or severely handicapped persons, or a selection of employers or firms that would hire this group of people”*

(53, male, Germany, employed, depression)

*“That there would be more job offers. Also that I could attend more job interviews”*

(43, female, Spain, unemployed, depression)

*“Psychological help in our workplace”*

(61, female, Greece, unemployed, DM)

“Support” can also arise from other persons at the workplace. Helpful aspects are support from colleagues, supervisors or human resource managers.

*“Employers, who would provide supported employment.”*

(38, male, Slovenia, unemployed, back and neck pain)

- “Attitude”: The theme “Attitude” was seen by the study participants as a need that could contribute to solving problems of the aforementioned stigmatization, discrimination and isolation. Most of the study participants wanted supervisors or colleagues to gain more knowledge about the health condition.

*“Understanding of chronic health conditions by supervisors and colleagues”*

(54, female, Germany, employed, depression)

*“Being recognized for my effort and my good work”*

(41, male, Spain, unemployed, depression)

### 3.3. Comparison of Factors Having a Negative or Positive Impact as Well as Needs by Occupational Status

**Figure 4:**
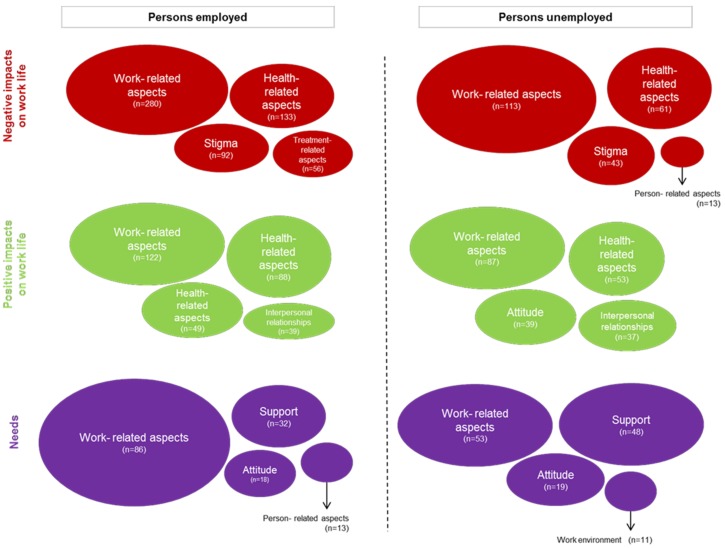
Themes stratified by the occupational status (employed and unemployed persons). The size of the circles is displayed according to the relative frequencies of the themes within the employed and unemployed persons, respectively.

Figure 4 displays the frequencies of themes stratified by the group of employed and unemployed persons regarding *Factors having a negative impact* and *Factors having a positive impact* on work life, as well as *Needs* experienced by persons with chronic health conditions. The relative frequencies of themes derived from the *Factors having a negative and positive impact* on work life are in the first place quite similar. Comparison of the *Needs* stratified by the occupational status also showed that the relative frequencies of themes are similar. For employed persons, one of the major *Needs* is to have more flexible contracts and work schedules and to have the possibility of changing the working position or changing from a full-time to a part-time contract. They want to see changes in the workplace and their work tasks with regards to their health condition.

*“Ability to better distribute the workload”*

(49, female, Italy, employed, back and neck pain)

*“Ergonomic chairs and support”*

(28, male, Italy, employed, back and neck pain)

The unemployed persons in our study sample are looking for workplaces that are also flexible and not burdensome. In addition, the combination of a partial pension with a part-time job is interesting for them. They want to be able to organize their daily life according to the fluctuations of their chronic health condition.

*“Flexible part-time position depending on the course of the disease”*

(54, female, Germany, unemployed, depression)

*“Being able to regulate working hours according to my illness”*

(38, male, Spain, unemployed, migraine/headache disorder; depression)

Study participants who were unemployed at the time of data collection expressed the Need for more support in searching for jobs or finding strategies for a better reintegration into employment.

*“Creation of programs for people with mental health problems aiming to help them find permanent jobs or long-term working contracts accompanied by support”*

(47, male, Greece, unemployed, depression)

*“Support in the job search in order to coordinate it”*

(50, female, Italy, unemployed, migraine/headache disorders)

Study participants who were in employment stressed the *Needs* of Finance-related and Support of others. In contrast study participants who were unemployed highlighted the *Need* for Service-related support. The first requested assistance from the authorities in order, for instance, to keep their business running or assistance at their workplace and more specifically support from supervisors or colleagues to reduce their workload in order to keep or return to work.

*“Something that could help me is more support from the state in order to be able to keep my business open”*

(62, female, Greece, employed, COPD)

*“I need more help within my work and certainly to be alleviated of my tasks”*

(34, female, Greece, employed, COPD)

## 4. Discussion

In this study we identified a variety of *Factors having a negative and positive impact* on work life, as well as *Needs* by getting first-hand evidence from persons with chronic health conditions in seven European countries. We found that the theme “Work-related aspects” such as career development, stress at the workplace, work structure and schedule as well as workload takes the first place in the *Factors having a negative and positive impact* on work life and *Needs*. The themes “Health-related aspects” and “Person-related aspects” are important themes as well when persons with chronic health conditions report on *Factors having a negative and positive impact* on their work lives. *Needs* are more focused on the themes “Work-related aspects”, “Support” and “Attitude”. Our results showed that *Factors having a negative and positive impact* on the work life as well as Needs slightly differ between employed and unemployed persons.

Comparing our results to previous research is challenging as most authors mainly analyzed factors that negatively impact the work life and focused on specific chronic health conditions [20,21,22]. In addition to that, most studies are single country studies. The qualitative study of Danielsson and colleagues identified categories of negative impacts of common mental health conditions as unipolar depression or anxiety on work life based on individual interviews. The study explored experiences of instability in work-related functioning [21]. The most relevant category having a negative impact was the core category “Working in dissonance”. Persons reported a disturbed workflow due to their mental health condition that influenced their social behavior. They distanced themselves from other people at work which caused them to experience isolation in the workplace. This is mainly in line with one of our results, namely the stigmatization of persons with chronic health conditions in the workplace.

In contrast to the studies mentioned above, in our study we obtained first-hand evidence on factors that have a positive impact as well as needs with regard to work life. The factors that have a positive impact gained from our study can be seen as a resource for people maintaining or returning to work. From our perspective, some of the factors found in our study which have a negative impact on work life can be seen as unmet needs of the persons with the six health conditions we focused on. To improve the work situation for people facing chronic health conditions we recommend focusing on factors that have a positive impact on work life as well as the needs expressed by the study participants. Some of the factors identified in our study that have a negative impact on work life can be used as tips to modify the work situation of persons with chronic health conditions. Strategies to improve the work situation of persons with chronic health conditions in Europe could focus on modification of work-related aspects such as the adaptation of working hours, working tasks and workload. In addition, support of others and attitude of others are relevant aspects in the social environment at the workplace and should be taken into account to improve the work life of persons with chronic health conditions.

Most of the work-related aspects identified in this study are modifiable (e.g., flexible working models, possibility of home office) and could be implemented in terms of strategies and policies at regional, national and European level. The results reaffirm the multidimensionality of the daily lives of individuals living with chronic health conditions and their long-term problems [23]. Most answers on the question on negative and positive impacts on work life as well as needs focused on the “Work-related aspects”. Earlier research on analyzing the differences between workers facing a chronic health condition and workers without a chronic health condition showed that workers with chronic health conditions experience more problems due to ageing, performance in work, more support needs and lower work ability scores [24]. Studies have shown that with increased pressure for labour market flexibility and increasing performance the idea have forced that working conditions in most European countries are related to a huge deterioration of psychological well-being and mental health [20,25]. According to the Finnish Institute of Occupational Health survey and the Netherlands Working Conditions survey, workers with chronic health conditions have little possibilities to influence their workload, working hours, working interface and working days [26]. Offering people the possibility of having flexible working conditions would probably foster the experience of having control over their working structure and would have a positive effect on health and well-being [27]. Up to now, there is limited evidence on the effect of strategies such as flexible working arrangements of people with chronic health conditions [26].

Our study also shed light on the importance of attitudes and support of colleagues and supervisors at the workplace. The study participants stressed the need to improve knowledge and understanding of chronic health conditions with respect to medical and work-related limitations and restrictions. Improving literacy on chronic health conditions at the workplace might reduce stigmatization and social exclusion often experienced by persons with chronic health conditions [28]. It is known that stigma as a social process leads to personal experiences of rejection, not being accepted or devaluation of persons with chronic health conditions combined with unreasonable social judgments [29,30]. Stigmatizing attitudes and ignorance of psychosocial workplace risks are leading causes on excluding workers from the labour market [31]. To overcome this change in the attitudes of supervisors and co-workers triggered by workshops providing general information about chronic health conditions and coping with these conditions would be a strategy to be thought about. A change in the attitudes of supervisors and co-workers could lead to more understanding and as a consequence to more support for colleagues facing chronic health conditions in work-related aspects. According to the European Network for Workplace Health Promotion (ENWHP), an implementation of health promotion offers an effective approach for improvement at the organizational level and working environment with active participation from employees with chronic health conditions [32]. Supporting employees with chronic health conditions and improving the interpersonal relationships in changing the attitude can lead to an improvement of productivity as well as reduction of costs such as the cost of sickness, work absence and staff turnover. 

Strengths of this study were the sampling scheme using the stakeholder list of the PATHWAYS project. The extensive data collection took place in seven European countries and focused on members of patient organizations. In selecting men and women from different age groups and different European countries, we provided a comprehensive and diverse opinion on persons’ needs. In this study, we followed a qualitative analysis of the open-ended questions and stressed the needs and factors that have a negative or positive impact on work lives of persons with chronic health conditions to hear first-hand experiences of these persons. We stratified the results taking into account the employment status at the level of themes derived from our qualitative analysis. We did not add other variables to quantitative analyses such as gender or study participants’ age. This could be done in future analysis to strengthen the final results of the PATHWAYS project.

This study has limitations worth to mention. The responsible person for each country translated the answers from mother tongue into English and sent it to the study coordinator. The translated answers were checked for intelligibility and in case of disagreement, a consultation with the responsible persons was held. However, loss of meaning caused by the translation into English cannot be completely excluded. We also noticed inconsistencies in the naming of chronic health conditions due to the self-reported diagnoses and were not able to provide information on clinically confirmed diagnoses. By providing a free text field participants of our study were allowed to name additional chronic health conditions besides the six health conditions we focused on in the PATHWAYS project. Our results showed that a majority of participants suffered from more than one health condition and comorbidities. This is in line with other studies; Camiciottoli and colleagues have reported in their study on COPD that about 80 percent suffer from at least one comorbidity [33]. Because of the large proportion of persons with comorbidities in our study population, we decided not to stratify our results taking into account the six chronic health conditions. Further studies should focus on condition-specific analyses taking into account the multi-morbidity of the chronic ill persons. Our results might also been affected by characteristics of the study population. Study participants from the different chronic health conditions differed in age, e.g., persons with COPD are generally older than persons with other health conditions considered in this study. However, we decided to report the results of this study without stratifying the results for the six chronic health conditions to allow for a sound consideration of needs experienced by persons with chronic health conditions in common. We also did not ask for condition-specific symptoms of the respective health condition and their effect on integration and reintegration at work. Because of the large proportion of persons with comorbidities in our study population, we decided not to stratify our results taking into account the six chronic health conditions.

## 5. Conclusions

This study identified factors that have a negative and positive impact on work life as well as needs of persons with chronic health conditions in seven European countries. Our study shed light on the importance of changing the attitudes of supervisors and co-workers to counteract the stigmatization of persons with chronic health conditions in the workplace. Work-related aspects such as career development, stress at the workplace, work structure and schedule as well as workload were also named by the participants as factors having a positive and negative impact on the work life. Our results stress the importance to adapt the workplace and environment according to the needs of persons facing chronic health conditions and to enable these persons to maintain a job or return to work in the long run. In light of an aging (work) population and an increase of chronic health conditions in European countries this will be one of the most important tasks employers and politicians have to take into account during the next decades. This study provides first-hand evidence for recommending strategies of integration and reintegration at work for persons with chronic health conditions in European countries.

## Figures and Tables

**Table 1:** Open-ended questions of the online survey (English template).

Open-Ended Questions
For persons employed at the time of data collection
Thinking about your chronic health condition what negatively impacts your work life the most?
Are there any other issues that negatively impact your work life?
Thinking about your chronic health condition what does positively impact your work life the most?
Are there any other issues that do positively impact your work life?
Do you have all you need to be able to work or to maintain work?
For persons not employed at the time of data collection
Thinking about your chronic health condition and the time you were at work/ employed what did negatively impact your work life the most?
Were there any other issues that did negatively impact your work life?
Thinking about your chronic health condition and the time you were at work/employed what did positively impact your work life the most?
Were there any other issues that did positively impact your work life?
Was there anything that would have been helpful to maintain your previous job?
Is there now anything that would be helpful to find a job?

**Table 2:** Characteristics of the study participants (*N* = 487).

Characteristics of the Study Participants	Descriptive Statistics
Age; median (range) (*N* = 487) *	46 (20–67)
Gender (female/male); *n* (%) (*N* = 485) *	335 (69.1%)/150 (30.9%)
Occupational status (employed/unemployed); *n* (%) (*N* = 487) *	294 (60.4%)/193 (39.6%)
Working sector (employed persons only); *n* (%) (*N* = 294) *	
Private sector	(52.7%)
Public sector	117 (39.8%)
Non-profit sector	22 (7.5%)
Occupation (employed persons only); *n* (%) (*N* = 291) *	
Professionals	94 (32.3%)
Clerical support workers	76 (26.1%)
Services and sales workers	30 (10.3%)
Technicians and associate professionals	29 (10.0%)
Managers	25 (8.6%)
Craft and related trades workers	14 (4.8%)
Elementary occupations	12 (4.1%)
Plant and machine operators and assemblers	8 (2.7%)
Skilled agricultural, forestry and fishery workers	3 (1.0%)
Chronic conditions; *n* (%) (N =487) **	
Back and neck pain	178 (36.6%)
Depression	140 (28.7%)
Diabetes mellitus	115 (23.6%)
Migraine/headache disorders	101 (20.7%)
Chronic obstructive pulmonary disorder	100 (20.5%)
Ischaemic heart disease	82 (16.8%)
Additional chronic health condition (e.g., anxiety disorders, arthritis, fibromyalgia, cancer) ***	231 (47.4%)

* Number of valid cases is displayed in parenthesis. ** Multiple answers possible. *** Other chronic health condition than the six selected for the study.

**Table 3:** Themes derived from *Factors having a negative impact* and *Factors having a positive impact* on work life as well as *Needs* to maintain a job or return to work marked with “x” in the respective column.

Themes	Negative Impact	Positive Impact	Needs
Work environment	x	x	x
Health-related aspects	x	x	x
Interpersonal relationships	x	x	x
Person-related aspects	x	x	x
Work-related aspects	x	x	x
Attitudes of others		x	x
Support of others		x	x
Treatment-related aspects	x		x
Job insecurity	x		
Stigma	x		
